# The Effect of 3′-Hydroxy-3,4,5,4′-Tetramethoxy -stilbene, the Metabolite of the Resveratrol Analogue DMU-212, on the Motility and Proliferation of Ovarian Cancer Cells

**DOI:** 10.3390/ijms21031100

**Published:** 2020-02-07

**Authors:** Andrzej Nowicki, Paulina Skupin-Mrugalska, Malgorzata Jozkowiak, Marcin Wierzchowski, Marcin Rucinski, Piotr Ramlau, Violetta Krajka-Kuzniak, Jadwiga Jodynis-Liebert, Hanna Piotrowska-Kempisty

**Affiliations:** 1 Department of Toxicology, Poznan University of Medical Sciences, Dojazd 30 St., PL-60-631 Poznan, Poland; andrzej.m.nowicki@gmail.com (A.N.); malgorzata.jozkowiak@gmail.com (M.J.); pioramlau@gmail.com (P.R.); liebert@ump.edu.pl (J.J.-L.); 2 Department of Inorganic and Analytical Chemistry, Poznan University of Medical Sciences, Grunwaldzka 6 St., 60-780 Poznan, Poland; psmrugalska@ump.edu.pl; 3 Department of Chemical Technology of Drugs, Poznan University of Medical Sciences, Grunwaldzka 6 St., PL-60-780 Poznan, Poland; mwierzch@ump.edu.pl; 4 Department of Histology and Embryology, Poznan University of Medical Sciences, Swiecickiego 6 St., PL-60-781 Poznan, Poland; marcinruc@ump.edu.pl; 5 Department of Pharmaceutical Biochemistry, Poznan University of Medical Sciences, Swiecickiego 4 St., 60-781 Poznan, Poland; vkrajka@ump.edu.pl

**Keywords:** resveratrol analogue, DMU-214, migration, proliferation, ovarian cancer

## Abstract

Targeting tumor cell motility and proliferation is an extremely important challenge in the prevention of metastasis and improving the effectiveness of cancer treatment. We recently published data revealing that DMU-214, the metabolite of firmly cytotoxic resveratrol analogue DMU-212, exerted significantly higher biological activity than the parent compound in ovarian cancer cells. The aim of the present study was to assess the molecular mechanism of the potential anti-migration and anti-proliferative effect of DMU-214 in ovarian cancer cell line SKOV-3. We showed that DMU-214 reduced the migratory capacity of SKOV-3 cells. The microarray analysis indicated ontology groups of genes involved in processes of negative regulation of cell motility and proliferation. Furthermore, we found DMU-214 triggered changes in expression of several migration- and proliferation-related genes (SMAD7, THBS1, IGFBP3, KLF4, Il6, ILA, SOX4, IL15, SRF, RGCC, GPR56) and proteins (GPR56, RGCC, SRF, SMAD7, THBS1), which have been shown to interact to each other to reduce cell proliferation and motility. Our study showed for the first time that DMU-214 displayed anti-migratory and anti-proliferative activity in SKOV-3 ovarian cancer cells. On the basis of whole transcriptome analysis of these cells, we provide new insight into the role of DMU-214 in inhibition of processes related to metastasis.

## 1. Introduction

Metastasis is the leading cause of death for patients diagnosed with cancer. Tumor invasiveness is known to be enhanced by epithelial-mesenchymal transition (EMT), which confers metastatic properties upon cancer cells by increasing their migration [1,2]. Therefore, targeting tumor cell motility is an extremely important and meaningful challenge in the prevention of metastasis and improving the effectiveness of cancer treatment.

Among the gynecological malignancies—those affecting the ovaries, uterus, and cervix—ovarian cancer displays the highest mortality rates. The overall five-year survival rate for patients with ovarian carcinoma is ~47%, which is mainly due to late diagnosis at an advanced stage with extensive metastasis in the peritoneal cavity. Ovarian cancer metastasizes either via direct extension from the primary tumor to surrounding tissue or when cancer cells detach from the extracellular matrix [3,4].

Resveratrol (3,4′,5-*trans*-trihydroxystilbene) is a phytoalexin produced in a variety of plants (e.g., grapes, peanuts, cranberries) as a result of pathogen attack or other environmental stressors [5]. The anti-migration and anti-proliferative effects of resveratrol has been shown in many cancer models [6,7,8]. Although resveratrol exerts strong anti-cancer activity, its clinical application is limited due to rapid biotransformation and elimination from the bloodstream [9]. The methylated resveratrol analogues have been revealed to show higher bioavailability and stronger cytotoxic effects as compared to the prototype compound [10,11,12,13]. 

**Figure 1:**
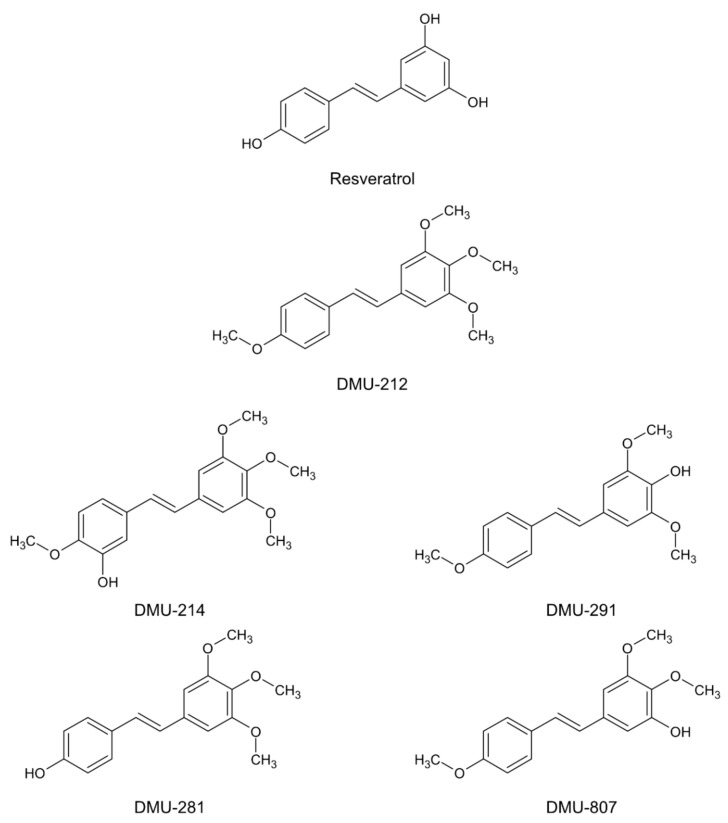
The chemical structures of resveratrol (the prototype compound), methylated resveratrol analogue (DMU-212) and its metabolites (DMU-214, DMU-291, DMU-281, DMU-807).

Among several methoxystilbenes studied, 3,4,5,4′-tetramethoxystilbene (DMU-212) seems to be one of the most potent drivers of cytotoxicity and apoptosis. DMU-212 has been shown to exert pro-apoptotic activity in several cancer cell lines, including transformed fibroblasts, liver, colon, hypopharynx, breast, prostate, and ovarian ones [14,15,16,17,18,19,20,21,22,23]. Biotransformation studies of DMU-212 have revealed its conversion to five metabolites: 3′-hydroxy-3,4,5,4′-tetramethoxystilbene (DMU-214), 4′-hydroxy-3,4,5-trimethoxystilbene (DMU-281), 4-hydroxy-3,5,4′-trimethoxystilbene (DMU-291), 4,4′-dihydroxy-3,5-dimethoxystilbene (DMU-295) and 3-hydroxy-4,5,4′-trimethoxystilbene (DMU-807) [17], (Figure 1). The cytotoxic activity of DMU-214 has been found to be significantly higher than that of the parent compound in ovarian cancer cells. Furthermore, DMU-214 has been shown to trigger G2/M cell cycle arrest and receptor-mediated apoptosis in the SKOV-3 ovarian cancer cell line lacking p53 [24]. 

The SKOV-3 cell line has been revealed to be resistant to many chemotherapeutics used routinely in ovarian cancer therapy [25,26]. Accordingly, our recently published results have shown lower cytotoxicity of DMU-214 in the SKOV-3 cell line as compared to other ovarian cancer cells [24]. However, the cytotoxic effect of this compound towards SKOV-3 was found to be similar, and even slightly higher than that of cisplatin [24,25]. Hence, DMU-214 might be suggested to inhibit the proliferation and dissemination of chemotherapy-resistant ovarian cancer cells. 

The aim of this study was to evaluate the molecular mechanism of the potential anti-metastatic activity of DMU-214 in human ovarian cancer cell line SKOV-3. The inhibition of cell motility was assayed by chemotaxis cell migration assay, RNA microarrays and the expression of proliferation- as well as motility- related genes and proteins.

## 2. Results

### 2.1. Effect of DMU-214 on Cell Migration 

**Figure 2:**
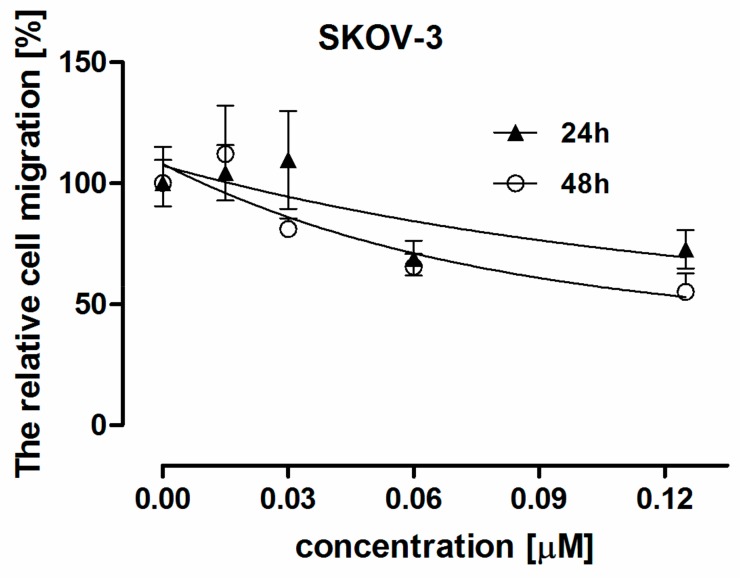
The cell migration after 12- and 24 h treatment of SKOV-3 cell line with DMU-214 at concentrations range 0.00–0.125 µM. The relative cell migration was determined by comparing to the control cells. The data are the mean ± SD.

The effect on cell migration was investigated after 12h and 24 h treatment of SKOV-3 cell line with DMU-214 (concentrations range 0.00–0.125 µM). As shown in Figure 2, treatment with the highest dose of DMU-214 (0.125 µM) for 12 and 24 h inhibited cell migration by ~45% and ~35%, respectively. Surprisingly, the decrease in cell motility by ~35% in a time-independent manner was found when SKOV-3 cells were treated with the compound tested at the concentration of 0.06 µM. We also observed the slightly reduced migratory capacity of SKOV-3 cells (~15%) when DMU-214 was applied at a concentration of 0.03 µM for 24 h. 

### 2.2. Microarray Analysis

**Figure 3:**
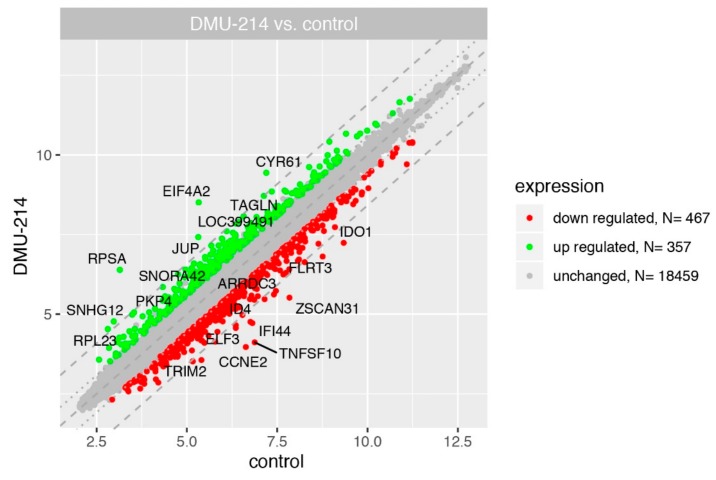
Total gene expression profile of the SKOV-3 cell line after 24 h incubation with DMU-214 (0.125 μM). Each dot represents a mean value of the single gene expression (*n* = 3/sample). Dotted lines indicate cut-off values (1.5-fold change in expression). The grey dots represent the genes below the cut-off limit (fold = 1.5 adjusted *p*-value < 0.05) considered as unchanged (*n* = 19228). The green colour marked the stimulated genes (*n* = 194) while the red ones inhibited by DMU-214 (*n* = 267). Ten genes with the highest and the lowest fold change values were labelled with the appropriate gene symbols.

In the current study, we used GeneChip Human Genome U219 microarray for simultaneous examination of the 19.285 human genes expression. The transcriptome study was performed 24 h after DMU-214 administration (0.125 μM) to the culture medium. The transcriptome profile was compared with the untreated (control) cells. The general profile of transcriptome regulation is shown in Figure 3 as a scatter plot. 

Differentially expressed genes (DEGs) were determined using the following selection criteria: an expression fold difference > absolute 1.5 and an adjusted *p*-value ≤ 0.05. In accordance with these criteria, 467 genes were down-regulated, and 357 were up-regulated as a consequence of DMU-214 action. The remaining 18459 genes did not meet the selection assumptions and were considered as unchanged genes.

**Figure 4:**
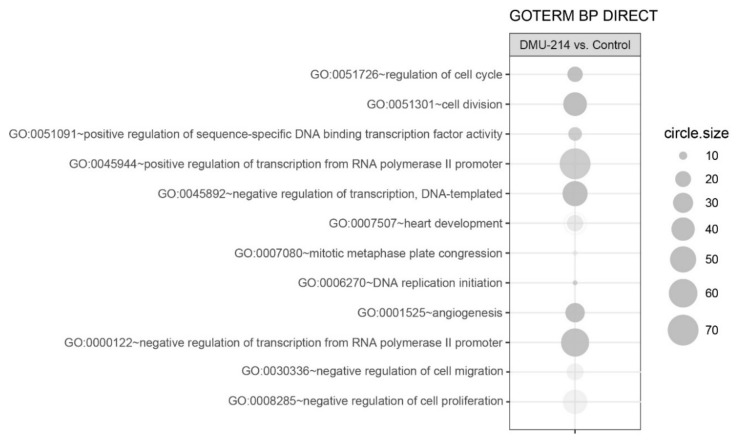
Bubble plot of overrepresented gene sets from DAVID GO BP DIRECT database. The graphs show only the GO groups above the established cut-off criteria (*p* with correction <0.05, a minimal number of genes per group >5). The size of each bubble reflects the number of differentially expressed genes assigned to the GO terms. The transparency of the bubbles displays the *p*-values (more transparent is closer to the border of *p* = 0.05).

In order to determine the biological processes regulated by DMU-214, we conducted an analysis of differentially expressed genes (DEGs) participation in specific ontological groups from the GO BP Direct database. The whole set of DEG consisting of 824 genes (467 down and 357 up-regulated) was functionally annotated and clustered using Database for Annotation, Visualization, and Integrated Discovery (DAVID) bioinformatics tools. The result of such analysis was shown as a bubble plot (Figure 4), in which only the ontological groups that met the following selection criteria were presented: *p* values below 0.05 and a minimum number of genes per group >5.

**Figure 5:**
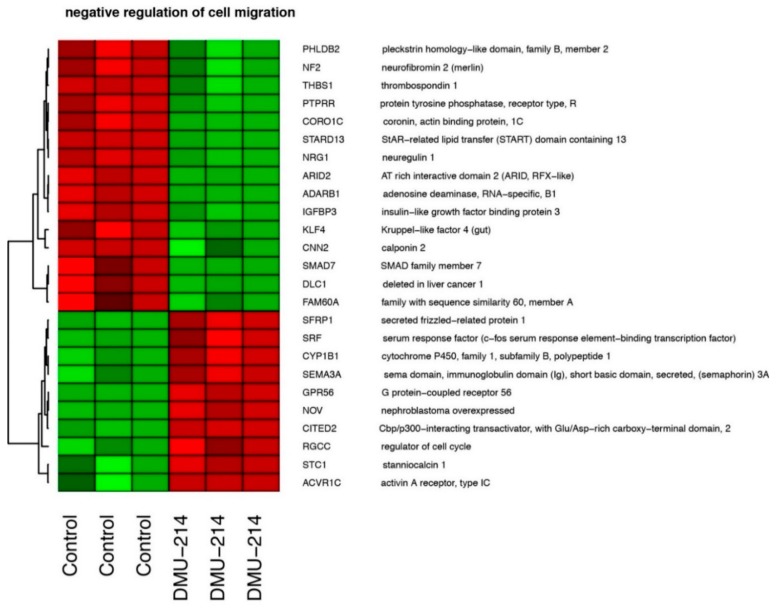
Heat map graph of differentially expressed genes belonging to “negative regulation of cell migration” GO BP term. Arbitrary signal intensity acquired from the microarray analysis is represented by colours (green—higher; red—lower expression in relation to control). Log2 signal intensity values for any single gene were resized to the Row Z-score scale. Gene symbols and gene names of differentially expressed genes were shown.

**Figure 6:**
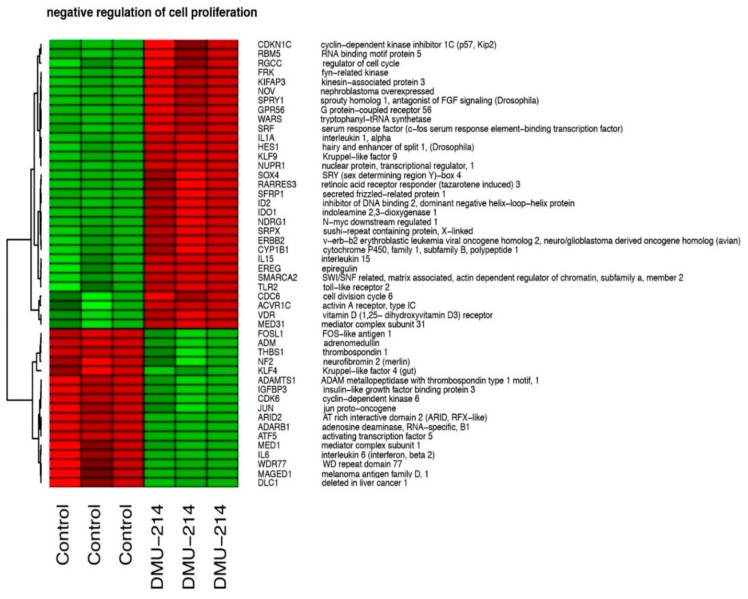
Heat map graph of differentially expressed genes belonging to “negative regulation of cell proliferation” GO BP term. Arbitrary signal intensity acquired from the microarray analysis is represented by colours (green—higher; red—lower expression in relation to control). Log2 signal intensity values for any single gene were resized to Row Z-score scale. Gene symbols and gene names of differentially expressed genes were shown.

In total, we identified 12 ontological groups formed by DEGs. They were involved in regulation of proliferation (*n* = 5), regulation of transcription (*n* = 5), and angiogenesis and heart development (*n* = 2). The detailed characteristics of the abovementioned ontological groups were as follows: “GO:0051726~regulation of cell cycle” (*n* = 19, adj. *p* value = 0.004), “GO:0051301~cell division” (*n* = 41, adj. *p* value = 3.59× 10^−5^), “GO:0051091~positive regulation of sequence-specific DNA binding transcription factor activity” (*n* = 16, adj. *p* value = 0.01), “GO:0045944~positive regulation of transcription from RNA polymerase II promoter” (*n* = 70, adj. *p* value = 0.02), “GO:0045892~negative regulation of transcription, DNA-templated” (*n* = 46, adj. *p* value = 0.002), “GO:0007507~heart development” (*n* = 21, adj. *p* value = 0.04), “GO:0007080~mitotic metaphase plate congression” (*n* = 9, adj. *p* value = 0.04), “GO:0006270~DNA replication initiation” (*n* = 9, adj. *p* value = 0.02), “GO:0001525~angiogenesis” (*n* = 28, adj. *p* value = 0.001), “GO:0000122~negative regulation of transcription from RNA polymerase II promoter” (*n* = 58, adj. *p* value = 0.004), “GO:0030336~negative regulation of cell migration“ (*n* = 25, adj. *p* value = 0.04), “GO:0008285~negative regulation of cell proliferation“ (*n* = 48, adj. *p* value = 0.04). Genes belonging to two ontological groups: “negative regulation of cell migration” and “negative regulation of cell proliferation” were clustered and visualised as heatmaps (Figure 5 and Figure 6, respectively). 

These groups include among others the following genes: serum response factor (c-fos serum response element-binding transcription factor)—SRF (fold= −1.8, *p* = 0.03); SMAD family member 7—SMAD7 (fold= 1.7, *p* = 0.01); G protein-coupled receptor 56—GPR56 (fold= −2.7, *p* = 0.005); insulin-like growth factor binding protein 3—IGFBP3 (fold = 1.5, *p*= 0.02); Kruppel-like factor 4—KLF4 (fold = 1.8, *p* = 0.02); interleukin 6—IL6 (fold = 1.8, *p* = 0.01); interleukin 1, alpha—IL1A (fold= −2.6, *p* = 0.02); interleukin 15—IL15 (fold = −1.5, *p*= 0.02); regulator of cell cycle—RGCC (fold= −1.62, *p* = 0.02); SRY (sex determining region Y)-box 4—SOX4 (fold= −2, *p* = 0.008), thrombospondin 1—THBS1 (fold = 1.6, *p* = 0.01).

**Figure 7:**
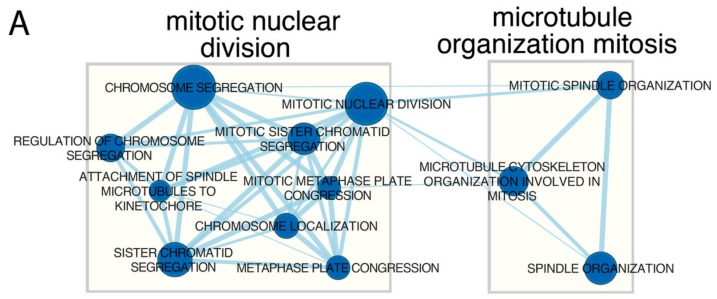

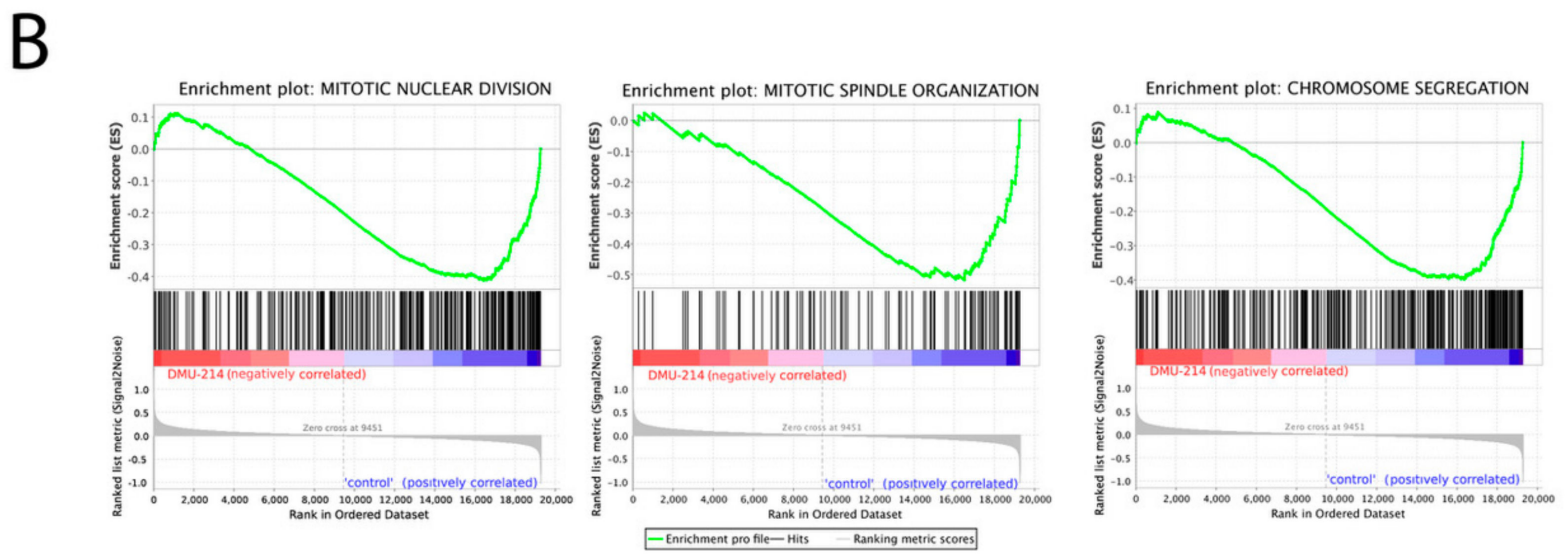
Gene set enrichment analysis. (**A**) Enrichment map of gene sets that were inhibited by DMU-214, from the Reactome database. The thickness of the connecting line corresponds to the level of gene matching to individual terms. (**B**) Enrichment plot of “Mitotic nuclear division”, “Mitotic spindle organization”, “Chromosome segregation”. Each of the graphs shows the value of the enrichment score (ES) indicating the depletion of genes forming these processes under the influence of DMU-214.

Gene set enrichment analysis (GSEA) confirms that DMU-214 is involved in the negative regulation of proliferation. We received two clusters of GSEA terms named “mitotic nuclear division” (*n* = 9) and “microtubule organisation mitosis” (*n* = 3), (Figure 7A). Three of GSEA terms that are related to mitotic cell division are presented as enrichment plot in Figure 7B. The majority of genes forming GSEA terms are negatively correlated suggesting their depletion under the influence of DMU-214. 

### 2.3. mRNA and Protein Expression Analysis

**Figure 8:**
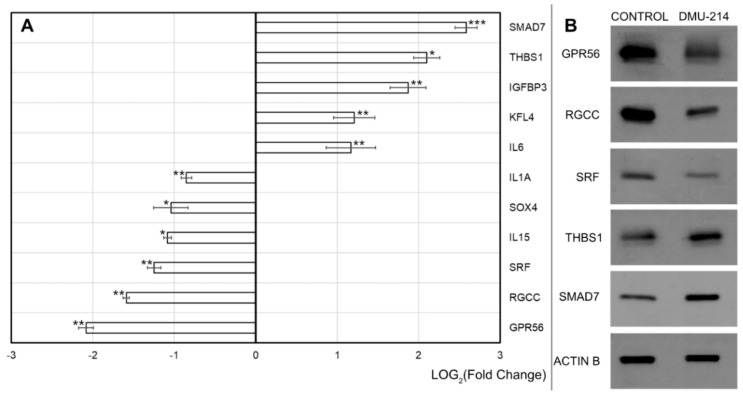
Effect of DMU-214 on migration- and proliferation-related genes and proteins in SKOV-3 cells. (**A**) Expression changes of SMAD7, THBS1, IGFBP3, KLF4, IL6, IL1A, SOX-4, IL15, SRF, RGCC and GPR56 genes in cells treated for 24 h with a vehicle or DMU-214 (0.125 μM). RT-qPCR validation of microarray results, presented in a form of a bar graph. Fold Change was presented in its logarithmic form to provide clear comparability of the results. Results of three replicates are presented as mean ± SD. ***p < 0.001, **p < 0.01 and *p < 0.05. (**B**) Changes of GPR56, RGCC, SRF, THBS1 and SMAD7 proteins level after treated cells for 24 h with a vehicle or DMU-214 (0.125 μM). Western blot was performed to validate microarray and RT-qPCR analysis.

The data obtained by means of the RNA microarray method were validated by RT-qPCR and Western blot for selected genes (Figure 8A) and proteins (Figure 8B), respectively. DMU-214 induced a marked up-regulation of mRNA expression of SMAD7, THBS1 and IGFBP3 in SKOV-3 cells ~6-, 5- and 3.5- fold, respectively (Figure 8A). A twofold increase in the expression of KLF4 and IL6 was also observed. Concurrently, DMU-214 decreased IL1A, IL15 and SOX4 transcripts level by ~50%. The most significant DMU-214-triggered effect was the down-regulation in mRNA expression of SRF, GPR56 and RGCC (~60–75%↓). 

As demonstrated in Western-blot analysis (Figure 8B), the levels of SRF, GPR56 and RGCC proteins expression paralleled those in transcripts. Exposure of SKOV-3 cells to DMU-214 resulted in an increased SMAD7 and THBS1 protein levels. However, the up-regulation of these proteins was less pronounced as compared to SMAD7 and THBS1 mRNA expression.

## 3. Discussion

The parent compound DMU-212 has been suggested to evoke anti-angiogenesis activity in vascular endothelial cell lines via the regulation of the migration- and angiogenesis-related genes expression pattern, however, the mechanism of action has not been clarified [27]. In the present study, we showed for the first time that DMU-214, the metabolite of DMU-212, inhibited the migratory capacity of SKOV-3 ovarian cancer cells. The compound tested was found to decrease the number of migrating cells significantly. Furthermore, the results of the microarray analysis pointed at the ontology group of genes involved in the negative regulation of cell motility and proliferation. Serum response factor (SRF) is a master regulator of several cellular processes including cancer cell growth, migration and angiogenesis [28]. We showed that DMU-214 down-regulated SRF mRNA and protein levels in the SKOV-3 ovarian cancer cell line. Chai et al. (2004) reported that knockdown of SRF protein in human and rat endothelial cells inhibited angiogenesis as well as impaired endothelial cell motility and proliferation [29]. These findings are consistent with our results since the down-regulation of SRF expression triggered by DMU-214 is accompanied by the decreased migration of SKOV-3 cells. Camoretti-Mercado et al. found that SRF interacts with SMAD 7, which is also known to inhibit cancer growth and metastasis [30]. The microarray analysis applied in the present study showed that DMU-214 increased SMAD 7 transcript level parallel to the protein level. Hence, the obtained results revealed the contribution of SMAD 7 and SRF in anti-migratory as well as anti-proliferative effects of the compound tested in the SKOV-3 ovarian cancer cell line. Furthermore, several other genes of negative regulation of cell motility pathway triggered by the compound tested – THBS1, GPR56, IGFBP3 and KLF4- have been revealed to be under transcriptional control of SRF [31,32,33,34]. 

THBS1, which is a member of the thrombospondin family, has been shown to be a natural inhibitor of proliferation, angiogenesis, and migration [35]. Traap et al. demonstrated the anti-migratory effects of resveratrol mediated by increased THBS1 transcript expression [36]. We confirmed these observations since the methylated resveratrol analogue DMU-214 caused an up-regulation of THBS1 mRNA and protein levels in SKOV-3 cells. Secord et al. showed lower expression of THBS1 in p53 mutant/null ovarian cancer cells, which have also been suggested to be less prone to chemotherapeutics [24,25,35]. Furthermore, the loss of wild-type (wt) p53 function in clinical cancer specimens of prostate, melanoma and ovary has been revealed to be manifested by the lower level of THBS1 expression and enhanced tumor dissemination [36]. Regardless of the reported relationship between decreased expression of THBS1, lack of p53 and increased metastasis, we showed in the current study the ability of DMU-214 to increase the expression of THBS1 followed by the inhibition of migration of SKOV-3 cells, which are commonly known to be deprived of wt p53. Hence, DMU-214 might be suggested to prevent ovarian cancer invasiveness and proliferation irrespective of the native level of p53. It seems to strengthen our previously reported findings that the lack of p53 protein in SKOV-3 cells did not abolish the anti-cancer activity of the compound tested [24]. 

GPR56, a member of the orphan G protein-coupled receptors (GPCRs) family, has been shown to be an oncogene in various types of cancer. Several studies have revealed that *GPR56* overexpression promoted the invasion of cancer cells, whereas depletion of *GPR56* remarkably inhibited cell proliferation, migration, and metastasis [37]. Our results are in agreement with the other authors’ findings since we showed that decreased expression of GPR56 triggered by DMU-214 was accompanied by suppression of motility of SKOV-3 cells. As more than 30% of anticancer drugs currently target GPCRs [37], the inhibition of GPR56 expression by DMU-214 seems to provide a promising approach to the treatment of cancer.

It is known that tumor invasiveness depends on the migratory and proliferative properties of cancer cells. We suggest that DMU-214 might effectively impair tumor dissemination since the results of microarray analysis also showed its effects on genes responsible for the negative regulation of SKOV-3 cells proliferation. We found that DMU-214 caused a significant increase in mRNA expression of IGFBP3 and KLF4, which have been reported to inhibit cancer cell migration as well as proliferation [33,38]. Interleukins (ILs) are commonly known to play a crucial role in many important to cancer biological processes, including cell proliferation. Rosenzweig et al. revealed that KLF4 modulates the expression of IL6, which has been found to inhibit breast cancer cell proliferation [39,40]. Our results are consistent with the previously reported role of KLF-4 since we observed that DMU-214 increased KLF4 as well as IL6 transcripts level in SKOV-3 ovarian cancer cells. Furthermore, our study showed the DMU-214-triggered decrease in the expression of IL1A and IL15, which are involved in enhancing cancer cell proliferation [41,42]. 

Our results of gene set enrichment analysis pointed at two clusters “mitotic nuclear division” and “microtubule organisation mitosis”, which confirmed the participation of DMU-214 in the negative regulation of cell proliferation. Cell proliferation is a strictly organized process that involves cell growth and cell division [43]. Regulator of Cell Cycle (RGCC) plays an important role in cell cycle regulation due to its ability to drive the G2/M phase transition [44]. Our previously published results indicated that DMU-214 caused a dramatic increase in the number of cells at G2/M [24]. Accordingly, in the present study, we found that DMU-214 decreased significantly RGCC mRNA and protein levels. It is in agreement with other authors’ findings showing that the cell cycle arrest at the G2/M phase in RGCC silenced cells has been linked with impaired proliferation [41]. Therefore, DMU-214 is suggested to exert anti-proliferative effects through the down-regulation of RGCC expression, which might be associated with the block of the cell cycle transition at the G2/M phase in SKOV-3 cell line shown in our previous study [24]. 

RGCC protein has been revealed to interact with SOX family transcription factors, which are involved in many biological processes, including proliferation. Some studies have reported that the principal role of SOX4 is related to the promotion of cancer cell proliferation [45]. In the present study, we observed a significantly decreased level of SOX4 mRNA in SKOV-3 cells treated with DMU-214. Hence, we suggest that the inhibited expression of both SOX4 and RGCC triggered by DMU-214 might also be involved in the mechanism of its anti-proliferative activity. 

It is well known that one of the main events involved in cancer invasion is cell migration as well as proliferation. Our study showed for the first time that DMU-214, the metabolite of DMU-212, displayed anti-migratory and anti-proliferative activity in SKOV-3 ovarian cancer cells lacking p53. Based on the whole transcriptome analysis of these cells, we provide new insight into the role of DMU-214 in regulation of processes related to metastasis.

## 4. Materials and Methods

### 4.1. Chemicals and Reagents

DMU-214 was synthesized as described elsewhere [21]. The identity and purity of each compound were confirmed by NMR and GC-mass spectroscopy. The QCM Chemotaxis Migration Assay was purchased from Merck (Darmstadt, Germany). The Affymetrix^®^ Human Genome U219 Array Strip was provided by Affymetrix (Santa Clara, CA, USA).

### 4.2. Cell Culture and Cell Migration Assays

SKOV-3 human ovarian cancer cell line was purchased from the European Type Culture Collection (Merck, Darmstadt, Germany), and maintained in phenol red-free DMEM medium Sigma-Aldrich Co. (St Louis, MO, USA) supplemented with 10% foetal bovine serum (FBS), 2 mM glutamine, penicillin (100 U/mL), and streptomycin (0.1 mg/mL) (Sigma-Aldrich Co., St. Louis, MO, USA). SKOV-3 cells were cultivated under standard conditions at 37 °C in a humidified atmosphere containing 5% CO_2_ and 95% air. To evaluate the effect of DMU-214 on SKOV-3 cells motility, the Chemotaxis Migration Assay (QCM), Merck (Darmstadt, Germany) was used according to the manufacturer’s protocol. Briefly, after incubation cells in a starving medium for 24 h, they were detached using the trypsin-EDTA solution and seeded (0.4 × 10^6^ cells/well in 250 µL of chemo-attractant-free medium containing 0.5% BSA) in inserts with different concentrations of DMU-214 that were placed into the lower chambers containing DMEM with 0.5% BSA. After 24 h incubation, inserts were transferred into new wells containing 400 µL of cell stain. Cotton swabs were used to remove any non-migratory cells from the interior of the inserts. Next, inserts were placed into clean wells containing 200 µL of extraction buffer. The optical density was measured at 560 nm using a BioTek reader (Winooski, VT, USA).

### 4.3. RNA Isolation

Total RNA isolation was performed using the modified Chomczynski method with the use of TRI Reagent (Sigma, St Louis, MO, USA) and RNeasy Mini Elute Cleanup Kit (Qiagen, Hilden, Germany), according to the manufacturer’s guidelines. The concentration of the total RNA was assessed spectrophotometrically by measuring absorbance (260 nm). The purity of the isolated RNA was determined by applying the 260/280 nm absorption ratio, which was equal or >1.8, as assumed (NanoDrop spectrophotometer, Thermo Scientific, Waltham, MA, USA). The quality and integrity of RNA were also verified in a Bioanalyzer 2100 (Agilent Technologies, Inc., Santa Clara, CA, USA). The resulting RNA integrity numbers (RINs) ranged from 8.5 to 10, with a mean of 9.2 (Agilent Technologies, Inc., Santa Clara, CA, USA). Each RNA sample was diluted to a concentration of 100 ng/μL.

### 4.4. Microarray Expression Analysis 

#### 4.4.1. Microarray Expression Experiment

The microarray study was carried out according to the procedure described earlier [46,47,48,49]. The previously isolated total RNA was pooled into four samples per each analysed groups, consisting of control and DMU-214 treated cells. The protocol involving transcription *in vitro*, biotin labelling and cDNA fragmentation for further hybridization was carried out using the Affymetrix GeneChip IVT Express Kit (Affymetrix). Obtained biotin-labelled fragments were hybridized with Affymterix GeneChip Human Genome U219 microarrays together with control cDNA and oligo B2. The hybridization process was conducted with the use of the AccuBlockTM Digital Dry Bath (Labnet International, Inc., Edison, NJ, USA) hybridization oven at 45 °C for 16 h. Then the microarrays were washed and stained according to the technical protocol using the Affymetrix GeneAtlas Fluidics Station. The array strips were scanned by the Imaging Station of GeneAtlas System. Preliminary analysis of the scanned chips was performed using Affymetrix GeneAtlas^TM^ Operating Software. The quality of gene expression data was verified using the quality control criteria established by the software. Obtained CEL files were imported into downstream data analysis.

#### 4.4.2. Microarray Data Analysis

All analyses were performed using BioConductor software with the relevant Bioconductor libraries, based on the statistical R programming language. The Robust Multiarray Average (RMA) normalization algorithm implemented in the “Affy” library was used for normalization, background correction, and calculation of the expression values of all of the examined genes [50]. The obtained *p*-value was corrected for multiple comparisons using the Benjamini and Hochberg false discovery rate. Biological annotation was taken from BioConductor “oligo” package where the annotated data frame object was merged with the normalized data set, leading to a complete gene data table [51]. Differential expression and statistical assessment were determined by applying the linear models for microarray data implemented in the “limma” library [52]. The selection criteria of a significantly changed gene expression were based on fold difference higher than absolute 1.5 and adjusted *p*-value <0.05. The result of such a selection was presented as a scatter plot, showing the total number of up- and down-regulated genes. 

#### 4.4.3. Assignment of Differentially Expressed Genes to Relevant Gene Ontology Biological Process (GO BP) Terms

The whole set of differentially expressed genes (DEGs) were subjected to functional annotation and clusterization using the Database for Annotation, Visualization, and Integrated Discovery (DAVID) bioinformatics tool [53]. Gene symbols of differentially expressed genes were uploaded to DAVID by the “RDAVIDWebService” BioConductor library [54], where DEGs were assigned to relevant GO terms, with subsequent selection of significantly enriched GO terms from GO BP Direct database. The *p*-values of selected GO terms were corrected using the Benjamini-Hochberg correction described as adjusted *p*-values. Relevant GO ontological groups with adjusted *p*-values below 0.05 and N per group >5 were visualized using a bubble plot. Gene expression values belonging to “negative regulation of cell migration” and “negative regulation of cell proliferation” ontological groups were subjected to hierarchical clustering and presented as heatmaps.

#### 4.4.4. Gene Set Enrichment Analysis (GSEA)

Gene Set Enrichment Analysis was used to determine enrichment or depletion in genes expression between two compared biological groups within a priori defined gene sets (GO terms, pathways). The method uses Kolmogorov–Smirnov (K-S) statistical test for the identification of significantly enriched or depleted groups of genes [55]. GSEA analysis has been conducted using GSEA Java Desktop Application from the Broad Institute (http://software.broadinstitute.org/gsea/index.jsp). Normalized data from all of the genes were transformed to an appropriate format and imported to application. Then, the predefined gene sets from the Reactome database (from the Molecular Signatures Database) were selected [56,57]. Genes belonging to the selected set were ranked according to the difference in their expression level using signal-to-noise ratio with 1000 times permutation. By walking down the ranked list of genes, the enrichment score (ES) was calculated for each selected gene set [58]. ESs were normalized by their gene set size, and false positives were corrected by FDR. Gene sets with adjusted *p* below 0.05 were exported to Cytoscape (ver. 3.7.2) [59], to generate links between significantly enriched processes in the form of Enrichment Map [60]. Enriched terms were clustered and annotated using the AutoAnnotate v1.3.2 Cytoscape plugin [61].

### 4.5. RT-qPCR

The expression levels of several selected genes were also validated with the aid of RT-qPCR (LightCycler^®^ Instrument 480 MultiwellPlate 96, Roche, Mannheim, Germany). Using specific primers and probes, the LightCycler^®^ 480 Probes Master kit was applied according to the manufacturer’s instructions. Specification of the reaction products was checked by determining the melting points (0.1°C/s transition rate).

### 4.6. Sodium Dodecyl Sulphate-Polyacrylamide Gel Electrophoresis (SDS-PAGE) and Western Blotting Analysis

SKOV-3 cells were treated with RIPA lysis buffer. Next, 30 μg of protein was resuspended in sample buffer and separated on 10% Tris-glycine gel using SDS-PAGE. Gel proteins were transferred to nitrocellulose, which was blocked with 5% milk in Tris-buffered saline/Tween. Immunodetection was performed with rabbit monoclonal anti-SRF Ab (#5147, Cell Signaling, Danvars, MA, USA), rabbit polyclonal anti-SMAD7 Ab (ab90086, Abcam, Cambridge, UK), rabbit polyclonal anti-THBS1 (ab85762, Abcam), rabbit polyclonal anti-GPR56 Ab (ab172361, Abcam) and rabbit polyclonal anti-RGC32 Ab (sc-84222, Santa Cruz Biotechnology Inc., Santa Cruz, CA, USA) followed by incubation with goat anti-rabbit HRP-conjugated Ab (#7074S, Cell Signaling). The membranes were also incubated with anti-actin HRP conjugated Ab (sc- 47778) to ensure equal protein loading of the lanes. Bands were revealed using SuperSignal West Femto maximum sensitivity substrate Pierce Biotechnology Inc. (Rockford, IL, USA).

### 4.7. Statistical Analyses

Data were expressed as means ± SD for three independent experiments. Statistical analysis was performed with one-way analysis of variance ANOVA followed by a Student-Newman-Keuls test. *p* values less than 0.05 were considered statistically significant.

## 5. Conclusions

It is well known that one of the main events involved in cancer invasion is cell migration as well as proliferation. Our study showed for the first time that DMU-214, the metabolite of DMU-212, displayed anti-migratory and anti-proliferative activity in SKOV-3 ovarian cancer cells lacking p53. Based on the whole transcriptome analysis of these cells, we provide new insight into the role of DMU-214 in regulation of processes related to metastasis.

## References

[1] Yeung T.L., Leung C.S., Yip K.P., Au Yeung C.L., Wong S.T., Mok S.C. (2015). Cellular and molecular processes in ovarian cancer metastasis. A Review in the Theme: Cell and Molecular Processes in Cancer Metastasis. Am. J. Physiol. Cell Physiol..

[2] Chen H., Nalbantoglu J. (2014). Ring cell migration assay identifies distinct effects of extracellular matrix proteins on cancer cell migration. BMC Res. Notes.

[3] Doubeni C.A., Doubeni A.R., Myers A.E. (2016). Diagnosis and Management of Ovarian Cancer. Am. Fam. Physician.

[4] Lengyel E. (2010). Ovarian cancer development and metastasis. Am. J. Pathol..

[5] Bavaresco L., Lucini L., Busconi M., Flamini R., De Rosso M. (2016). Wine Resveratrol: From the Ground Up. Nutrients.

[6] LaFoya B., Munroe J.A., Albig A.R. (2019). A comparison of resveratrol and other polyphenolic compounds on Notch activation and endothelial cell activity. PLoS ONE.

[7] Yang S., Li W., Sun H., Wu B., Ji F., Sun T., Chang H., Shen P., Wang Y., Zhou D. (2015). Resveratrol elicits anti-colorectal cancer effect by activating miR-34c-KITLG in vitro and in vivo. BMC Cancer.

[8] Han Y., Jo H., Cho J., Dhanasekaran D.N., Song Y.S. (2019). Resveratrol as a Tumor-Suppressive Nutraceutical Modulating Tumor Microenvironment and Malignant Behaviors of Cancer. Int. J. Mol. Sci..

[9] Chimento A., De Amicis F., Sirianni R., Sinicropi M.S., Puoci F., Casaburi I., Saturnino C., Pezzi V. (2019). Progress to Improve Oral Bioavailability and Beneficial Effects of Resveratrol. Int. J. Mol. Sci..

[10] Van den Brand A.D., Villevoye J., Nijmeijer S.M., van den Berg M., van Duursen M.B.M. (2019). Anti-tumor properties of methoxylated analogues of resveratrol in malignant MCF-7 but not in non-tumorigenic MCF-10A mammary epithelial cell lines. Toxicology.

[11] Li H., Wu W.K., Li Z.J., Chan K.M., Wong C.C., Ye C.G., Yu L., Sung J.J., Cho C.H., Wang M. (2010). 2,3′,4,4′,5′-Pentamethoxytrans- stilbene, a resveratrol derivative, inhibits colitis-associated colorectal carcinogenesis in mice. Br. J. Pharmacol..

[12] Tsai H.Y., Ho C.T., Chen Y.K. (2017). Biological actions and molecular effects of resveratrol, pterostilbene, and 3′-hydroxypterostilbene. J. Food Drug Anal..

[13] Fulda S. (2010). Resveratrol and derivatives for the prevention and treatment of cancer. Drug Discov. Today.

[14] Gosslau A., Chen M., Ho C.T., Chen K.Y. (2005). A methoxy derivative of resveratrol analogue selectively induced activation of the mitochondrial apoptotic pathway in transformed fibroblasts. Br. J. Cancer.

[15] Ma Z., Molavi O., Haddadi A., Lai R., Gossage R.A., Lavasanifar A. (2008). Resveratrol analog trans 3,4,5,4′-tetramethoxystilbene (DMU-212) mediates anti-tumor effects via mechanism different from that of resveratrol. Cancer Chemother. Pharmacol..

[16] Sale S., Tunstall R.G., Ruparelia K.C., Potter G.A., Steward W.P., Gescher A.J. (2005). Comparison of the effects of the chemopreventive agent resveratrol and its synthetic analog trans 3,4,5,4′-tetramethoxystilbene (DMU-212) on adenoma development in the Apc(Min+) mouse and cyclooxygenase-2 in human-derived colon cancer cells. Int. J. Cancer.

[17] Sale S., Verschoyle R.D., Boocock D., Jones D.J., Wilsher N., Ruparelia K.C., Potter G.A., Farmer P.B., Steward W.P., Gescher A.J. (2004). Pharmacokinetics in mice and growthinhibitory properties of the putative cancer chemopreventive agent resveratrol and the synthetic analogue trans 3,4,5,4′-tetramethoxystilbene. Br. J. Cancer.

[18] Piotrowska H., Myszkowski K., Ziółkowska A., Kulcenty K., Wierzchowski M., Kaczmarek M., Murias M., Kwiatkowska-Borowczyk E., Jodynis-Liebert J. (2012). Resveratrol analogue 3,4,4′,5-tetramethoxystilbene inhibits growth, arrests cell cycle and induces apoptosis in ovarian SKOV-3 and A- 2780 cancer cells. Toxicol Appl. Pharmacol..

[19] Piotrowska H., Myszkowski K., Abraszek J., Kwiatkowska-Borowczyk E., Amarowicz R., Murias M., Wierzchowski M., Jodynis-Liebert J. (2014). DMU-212 inhibits tumor growth in xenograft model of human ovarian cancer. Biomed. Pharmacother..

[20] Piotrowska H., Myszkowski K., Amarowicz R., Murias M., Kulcenty K., Wierzchowski M., Jodynis-Liebert J. (2013). Different susceptibility of colon cancer DLD-1 and LOVO cell lines to apoptosis induced by DMU-212, a synthetic resveratrol analogue. Toxicol. In Vitro.

[21] Androutsopoulos V.P., Ruparelia K.C., Papakyriakou A., Filippakis H., Tsatsakis A.M., Spandidos D.A. (2011). Anticancer effects of the metabolic products of the resveratrol analogue, DMU-212: Structural requirements for potency. Eur. J. Med. Chem..

[22] Kleszcz R., Paluszczak J., Krajka-Kuźniak V., Baer-Dubowska W. (2018). The inhibition of c-MYC transcription factor modulates the expression of glycolytic and glutaminolytic enzymes in FaDu hypopharyngeal carcinoma cells. Adv. Clin. Exp. Med..

[23] Pastorková B., Vrzalová A., Bachleda P., Dvořák Z. (2017). Hydroxystilbenes and methoxystilbenes activate human aryl hydrocarbon receptor and induce CYP1A genes in human hepatoma cells and human hepatocytes. Food Chem. Toxicol..

[24] Piotrowska-Kempisty H., Ruciński M., Borys S., Kucińska M., Kaczmarek M., Zawierucha P., Wierzchowski M., Łażewski D., Murias M., Jodynis-Liebert J. (2016). 3′-hydroxy-3,4,5,4′-tetramethoxystilbene, the metabolite of resveratrol analogue DMU-212, inhibits ovarian cancer cell growth in vitro and in a mice xenograft model. Sci. Rep..

[25] Yellepeddi V.K., Kumar A., Maher D.M., Chauhan S.C., Vangara K.K., Palakurthi S. (2011). Biotinylated PAMAM dendrimers for intracellular delivery of cisplatin to ovarian cancer: Role of SMVT. Anticancer Res..

[26] Wahl H., Tan L., Griffith K., Choi M., Liu J.R. (2007). Curcumin enhances Apo2L/TRAIL-induced apoptosis in chemoresistant ovarian cancer cells. Gynecol. Oncol..

[27] Miao Y., Cui L., Chen Z., Zhang L. (2016). Gene expression profiling of DMU-212-induced apoptosis and anti-angiogenesis in vascular endothelial cells. Pharm. Biol..

[28] Franco C.A., Li Z. (2009). SRF in angiogenesis. Branching the vascular system. Cell Adhr. Migr..

[29] Chai J., Jones M.K., Tarnawski A.S. (2004). Serum response factor is a critical requirement for VEGF signaling in endothelial cells and VEGF-induced angiogenesis. FASEB J..

[30] Camoretti-Mercado B., Fernandes D.J., Dewundara S., Churchill J., Ma L., Kogut P.C., McConville J.F., Parmacek M.S., Solway J. (2006). Inhibition of transforming growth factor beta-enhanced serum response factor-dependent transcription by SMAD7. J. Biol. Chem..

[31] Weinl C., Riehle H., Park D., Stritt C., Beck S., Huber G., Wolburg H., Olson E.N., Seeliger M.W., Adams R.H. (2013). Endothelial SRF/MRTF ablation causes vascular disease phenotypes in murine retinae. J. Clin. Invest..

[32] Kishore A., Purcell R.H., Nassiri-Toosi Z., Hall R.A. (2016). Stalk-dependent and Stalk-independent Signaling by the Adhesion G Protein-coupled Receptors GPR56 (ADGRG1) and BAI1 (ADGRB1). J. Biol. Chem..

[33] Kim J.H., Choi D.S., Lee O.H., Oh S.H., Lippman S.M., Lee H.Y. (2011). Antiangiogenic antitumor activities of IGFBP-3 are mediated by IGF-independent suppression of Erk1/2 activation and Egr-1-mediated transcriptional events. Blood.

[34] Deaton R.A., Gan Q., Owens G.K. (2009). Sp1-dependent activation of KLF4 is required for PDGF-BB-induced phenotypic modulation of smooth muscle. Am. J. Physiol. Heart Circ. Physiol..

[35] Secord A.A., Bernardini M.Q., Broadwater G., Grace L.A., Huang Z., Baba T., Kondoh E., Sfakianos G., Havrilesky L.J., Murphy S.K. (2013). TP53 status is associated with thrombospondin1 expression in vitro. Front. Oncol..

[36] Trapp V., Parmakhtiar B., Papazian V., Willmott L., Fruehauf J.P. (2010). Anti-angiogenic effects of resveratrol mediated by decreased VEGF and increased TSP1 expression in melanoma-endothelial cell co-culture. Angiogenesis.

[37] Ji B., Feng Y., Sun Y., Ji D., Qian W., Zhang Z., Wang Q., Zhang Y., Zhang C., Sun Y. (2018). GPR56 promotes proliferation of colorectal cancer cells and enhances metastasis via epithelial-mesenchymal transition through PI3K/AKT signaling activation. Oncol. Rep..

[38] Fan Y., Lu H., Liang W., Hu W., Zhang J., Chen Y.E. (2017). Krüppel-like factors and vascular wall homeostasis. J. Mol. Cell Biol..

[39] Rosenzweig J.M., Glenn J.D., Calabresi P.A., Whartenby K.A. (2013). KLF4 modulates expression of IL-6 in dendritic cells via both promoter activation and epigenetic modification. J. Biol. Chem..

[40] Badache A., Hynes N.E. (2001). Interleukin 6 inhibits proliferation and, in cooperation with an epidermal growth factor receptor autocrine loop, increases migration of T47D breast cancer cells. Cancer Res..

[41] Maund S.L., Barclay W.W., Hover L.D., Axanova L.S., Sui G., Hipp J.D., Fleet J.C., Thorburn A., Cramer S.D. (2011). Interleukin-1 alpha mediates the anti-proliferative effects of 1,25 dihydroxyvitamin D3 in prostate progenitor/stem cells. Cancer Res..

[42] Van Acker H.H., Anguille S., Willemen Y., Van den Bergh J.M., Berneman Z.N., Lion E., Smits E.L., Van Tendeloo V.F. (2016). Interleukin-15 enhances the proliferation, stimulatory phenotype, and antitumor effector functions of human gamma delta T cells. J. Hematol. Oncol..

[43] Dolatabadi S., Candia J., Akrap N., Vannas C., Tesan Tomic T., Losert W., Landberg G., Åman P., Ståhlberg A. (2017). Cell Cycle and Cell Size Dependent Gene Expression Reveals Distinct Subpopulations at Single-Cell Level. Front. Genet..

[44] Shen Y.L., Liu H.J., Sun L., Niu X.L., Kuang X.Y., Wang P., Hao S., Huang W.Y. (2016). Response gene to complement 32 regulates the G2/M phase checkpoint during renal tubular epithelial cell repair. Cell Mol. Biol. Lett..

[45] Chen D., Hu C., Wen G., Yang Q., Zhang C., Yang H. (2018). Down regulated SOX4 expression suppresses cell proliferation, migration, and induces apoptosis in osteosarcoma in vitro and in vivo. Calcif. Tissue Int..

[46] Szyszka M., Paschke L., Tyczewska M., Jopek K., Celichowski P., Milecka P. (2019). Analysis of Transcriptome, Selected Intracellular Signaling Pathways, Proliferation and Apoptosis of LNCaP Cells Exposed to High Leptin Concentrations. Int. J. Mol. Sci..

[47] Jopek K., Tyczewska M., Ramanjaneya M., Szyszka M., Celichowski P., Milecka P., Malendowicz L.K., Rucinski M. (2018). Effect of ACTH and hCG on the Expression of Gonadotropin-Inducible Ovarian Transcription Factor 1 (Giot1) Gene in the Rat Adrenal Gland. Int. J. Mol. Sci..

[48] Jopek K., Tyczewska M., Celichowski P., Malendowicz L.K., Rucinski M. (2018). Transcriptome Profile in Unilateral Adrenalectomy-Induced Compensatory Adrenal Growth in the Rat. Int. J. Mol. Sci..

[49] Jopek K., Celichowski P., Szyszka M., Tyczewska M., Milecka P., Malendowicz L.K. (2017). Transcriptome Profile of Rat Adrenal Evoked by Gonadectomy and Testosterone or Estradiol Replacement. Front. Endocrinol. (Lausanne).

[50] Gautier L., Cope L., Bolstad B.M., Irizarry R.A. (2004). affy--analysis of Affymetrix GeneChip data at the probe level. Bioinformatics.

[51] Carvalho B.S., Irizarry R.A. (2010). A framework for oligonucleotide microarray preprocessing. Bioinformatics.

[52] Ritchie M.E., Phipson B., Wu D., Hu Y., Law C.W., Shi W., Smyth G.K. (2015). limma powers differential expression analyses for RNA-sequencing and microarray studies. Nucleic Acids Res..

[53] Dennis G., Sherman B.T., Hosack D.A., Yang J., Gao W., Lane H.C., Lempicki R.A. (2003). DAVID: Database for Annotation, Visualization, and Integrated Discovery. Genome Biol..

[54] Fresno C., Fernandez E.A. (2013). RDAVIDWebService: A versatile R interface to DAVID. Bioinformatics.

[55] Damian D., Gorfine M. (2004). Statistical concerns about the GSEA procedure. Nat. Genet..

[56] Liberzon A., Birger C., Thorvaldsdottir H., Ghandi M., Mesirov J.P., Tamayo P. (2015). The Molecular Signatures Database (MSigDB) hallmark gene set collection. Cell Syst..

[57] Jassal B., Matthews L., Viteri G., Gong C., Lorente P., Fabregat A., Sidiropoulos K., Cook J., Gillespie M., Haw R. (2020). The reactome pathway knowledgebase. Nucleic Acids Res..

[58] Subramanian A., Tamayo P., Mootha V.K., Mukherjee S., Ebert B.L., Gillette M.A., Paulovich A., Pomeroy S.L., Golub T.R., Lander E.S. (2005). Gene set enrichment analysis: A knowledge-based approach for interpreting genome-wide expression profiles. Proc. Natl. Acad. Sci. USA.

[59] Shannon P., Markiel A., Ozier O., Baliga N.S., Wang J.T., Ramage D., Amin N., Schwikowski B., Ideker T. (2003). Cytoscape: A software environment for integrated models of biomolecular interaction networks. Genome Res..

[60] Merico D., Isserlin R., Steuker O., Emili A., Bader G.D. (2010). Enrichment map: A network-based method for gene-set enrichment visualization and interpretation. PLoS ONE.

[61] Kucera M., Isserlin R., Arkhangorodsky A., Bader G.D. (2016). AutoAnnotate: A Cytoscape app for summarizing networks with semantic annotations. F1000Res.

